# Response to Weng

**DOI:** 10.14309/ctg.0000000000000383

**Published:** 2021-07-12

**Authors:** Andrew S. Allegretti

**Affiliations:** 1 Division of Nephrology, Department of Medicine, Massachusetts General Hospital, Boston, Massachusetts, USA.

We thank Dr. Weng for continuing the dialog on the use of neutrophil gelatinase-associated lipocalin (NGAL) in the diagnosis and prognosis of acute kidney injury (AKI) and cirrhosis (1). The primary aim of our prospective cohort study was to validate findings from 2 separate cohorts from European investigators around the use of urinary NGAL in AKI and decompensated cirrhosis. In the first cohort, urinary NGAL differentiated structural kidney injury (e.g., acute tubular necrosis [ATN]) from functional kidney injury (e.g., prerenal AKI and hepatorenal syndrome [HRS]) in a population of cirrhotic patients admitted with AKI (2). In the second, urinary NGAL was associated with mortality across a more diverse spectrum of acute on chronic liver failure (ACLF), which included hospitalized patients with and without AKI (3). In particular, NGAL seems to hold greater prognostic significance at higher model for end-stage liver disease scores. We confirmed both of these observations in our study (4), which we believe contributes to the literature by increasing the generalizability of these results through an American cohort.

Despite these encouraging findings, Dr Weng highlights several limitations when studying this population that warrant further discussion. First, differentiating the type of AKI remains an enormous clinical challenge in cirrhosis. Hemodynamic AKI in cirrhosis has historically been divided into 3 main subgroups in the literature: prerenal AKI, HRS, and ATN (5,6). We believe this differentiation to be extremely important because management strategies differ for each cause: Prerenal AKI is treated with volume resuscitation, HRS warrants a combination of judicious volume administration and splanchnic vasoconstrictors, and ATN is treated with supportive measures. All 3 of these AKI subtypes represent clinical diagnoses and thus require nuanced and attentive assimilation of objective tests, subjective findings, and application of diagnostic criteria to define them. To complicate matters further, these syndromes can overlap and evolve, requiring an iterative approach to the diagnosis of AKI in cirrhosis (7). We believe we applied a logical set of definitions to categorize type of AKI in our study, which resulted from a series of discussions between the hepatologists and nephrologists in our multicenter collaborative. We identified HRS using the widely accepted Ascites Club criteria (7). Prerenal AKI was defined as prompt resolution of AKI with volume administration in a patient with a clinical history supportive of volume loss. In cirrhosis, ATN lacks an accepted objective set of clinical criteria. Although attempts have been made to apply fractional sodium excretion, urinary osmolality, and urinary sediment findings to aid in diagnosis (2), we did not believe such criteria were widely accepted or validated enough to include in our definition and thus used a clinical history of ischemia or nephrotoxin exposure along with a lack of improvement after volume administration to define ATN. In their 2011 study, Martin-Llahi et al. identified a fourth subgroup of AKI: renal failure associated with infection (8). We would discourage use of this category for 2 reasons: (i) Infections can be triggers of prerenal AKI, HRS, and ATN alike, and (ii) presence of an infection does not inform kidney-specific management. Although defining types of AKI in cirrhosis is complicated, one thing is clear—a strong consensus on definitions for non-HRS subgroups of AKI would move research in this space forward tremendously.

We agree that there are limitations in interpreting the performance of NGAL as a prognostic marker of mortality because of nonconsecutive enrollment of our study population, although data thus far have been promising. One must also consider the availability of this assay because urinary NGAL is not currently approved for use in the United States. A multicenter, prospective study design that rigorously interrogates markers such as NGAL in a predefined group of decompensated cirrhotic patients would be the logical next step in improving our ability to prognosticate in this vulnerable population.

## CONFLICTS OF INTEREST

**Guarantor of the article:** Andrew S. Allegretti, MD, MSc.

**Specific author contributions:** A.S.A. wrote this article. Members of the HRS-HARMONY study group read and approved this article.

**Financial support:** A.S.A. is supported by American Heart Association award 18CDA34110131.

**Potential competing interests:** A.S.A. has served on scientific advisory boards for Mallinckrodt Pharmaceuticals and consulted for Cymabay Therapeutics.
